# A Rare Mimicker: Renal Endometriosis and Its Diagnostic Pitfalls—Review of Reported Cases

**DOI:** 10.3390/diagnostics16162535

**Published:** 2026-08-11

**Authors:** Nebojsa Zecevic, Ana Tomic, Marija Rovcanin, Ana Mladenovic Markovic, Svetlana Jankovic

**Affiliations:** 1 Clinic for Gynecology and Obstetrics, Narodni Front, Kraljice Natalije 62, 11000 Belgrade, Serbia; 2 Faculty of Medicine, University of Belgrade, Dr Subotica Starijeg 8, 11000 Belgrade, Serbia; 3 Center for Radiology, University Clinical Center of Serbia, Pasterova 2, 11000 Belgrade, Serbia

**Keywords:** endometriosis, kidney, renal, CT, histopathology, nephrectomy

## Abstract

**Background/Objectives**: Primary renal endometriosis with extrapelvic locations of endometriosis lesions is deemed extremely rare. Therefore, the aim of this review was to assess the current literature on renal endometriosis, encompassing a summary of recorded living-patient cases, imaging and pathological features, chosen therapeutic approaches, and patient outcomes. **Methods**: We performed a narrative review with a systematic identification and presentation of published cases of histopathologically proven kidney endometriosis. A literature search was carried out across PubMed, Scopus, and Web of Science for relevant studies, i.e., case reports and series that met predefined inclusion and exclusion criteria specified by the modified PECOS framework. **Results**: A total of 19 publications reporting a total of 20 reported cases were included in the in-depth analysis. The noted condition affected women of reproductive age, with a mean age of 37.4, with the most common presenting symptoms being lumbar or flank pain, gross hematuria, abdominal pain, and tenderness. Most were single-mass lesions detected on the right kidney, with a mean largest diameter of 6.7 cm. Most were CT-characterized as either hyperattenuating lesions, septate lesions with foci of calcifications and soft tissue components, or areas of necrosis or cystic change. As CT presented features that would not allow the exclusion of malignant kidney tumors, most patients were treated surgically with nephrectomy. Diagnosis was confirmed by histopathology of recorded endometrial glands and stroma. **Conclusions**: The diagnosis of these lesions is exceptionally challenging because of their nonspecific symptoms, the rare association with the menstrual cycle, the rarity of dysmenorrhea, and the occasional coexistence of ovarian endometriosis, with no distinct endometriosis-specific clinical and imaging pattern emerging.

## 1. Introduction

Endometriosis is a common benign gynecological condition defined as the presence of endometrial tissue (endometrial glands and stroma) outside the uterine cavity.

The development of endometriosis lesions can be explained by a number of theories, including the process of retrograde menstruation, possible hematogenous or lymphatic dissemination, and other hormonal, inflammatory, or immunologic environment-related factors that may influence the implantation and persistence of endometrial cells and tissue. Alternatively, local tissue derived from the remains of fetal embryonic cells or from the genitourinary system may undergo aberrant differentiation, potentially resulting in endometriosis lesions [1,2].

Endometriosis is believed to impact the wellbeing of more than 190 million women [3], affecting approximately 10% of all women of reproductive age [4]. This is probably an underestimate as some individuals may have lesions that remain undiagnosed [3]. Ectopic tissue is predominantly situated in the pelvis and surrounding peritoneum [5]. However, extrapelvic endometriosis, traditionally thought to be rare, has been reported in a considerable number of cases [6]. The occurrence of urinary tract endometriosis is approximated to range from 0.3 to 12% among all individuals with endometriosis [7], with the urine system ranking as the second most frequent location behind the gastrointestinal tract [8]. Bladder and ureteral involvement are the most frequently documented, with 70–85% of urinary tract endometriosis cases affecting the bladder [9]. The kidney is typically secondary for endometriotic lesions in the bladder or ureter, resulting in hydroureteronephrosis, obstructive uropathy [10], and diminished renal function [11,12,13], along with nonspecific symptoms such as back pain [12] and renal colic [13].

However, primary renal endometriosis is deemed extremely rare [14]. Although uncommon, kidney endometriosis was initially histopathologically characterized by Marshal et al. in 1943 [15]. According to Hajdu and Koss [16], several incidences were documented in the latter part of the 20th century, and by 1970, there were seven cases, with diagnoses confirmed after nephrectomy in five occurrences and discovered incidentally after autopsy in two cases.

Nonetheless, this entity lacks widespread acknowledgment or scrutiny, as endometriotic lesions in the bladder and ureters are more common and recorded in scholarly works. There are no extensive studies that highlight the significance of this condition concerning kidney function, particularly in light of advancements in diagnostic techniques such as CT (computed tomography) and MRI (magnetic resonance imaging). The objective of this review was to evaluate the existing literature on renal endometriosis, including a summary of documented cases involving living patients, diagnostic methods employed, imaging and pathological characteristics, impacts on renal function, selected treatment strategies, and patient outcomes related to this condition.

## 2. Materials and Methods

### 2.1. Search Strategy and Data Screening

This review employs a narrative, unsystematic methodology to analyze and synthesize the available literature on the specified topic, offering a comprehensive, although non-exhaustive, summary of essential concepts, trends, and conclusions from pertinent sources. A literature search was conducted using PubMed, Scopus, and Web of Science to find all pertinent studies. Keywords and phrases associated with “endometriosis,” “renal,” and “kidney” were employed to locate pertinent papers. Filters were utilized to select only complete studies published in English that detail cases of endometriosis impacting kidney tissue, including documented clinical and diagnostic protocols that resulted in a definitive, histopathologically confirmed diagnosis. The exclusion criteria encompassed papers that were not fully accessible or were published in languages other than English, as well as those that addressed secondary renal impairment in instances of endometriosis impacting other regions of the urinary tract. The study did not include any cases diagnosed postmortem. Given the infrequency of the condition, permissible study designs included case reports and case series. Titles and abstracts were initially evaluated, and possibly pertinent papers were identified and assessed for eligibility criteria. The last search was performed on 30 March 2026.

### 2.2. Criteria for Qualification

**Figure 1:**
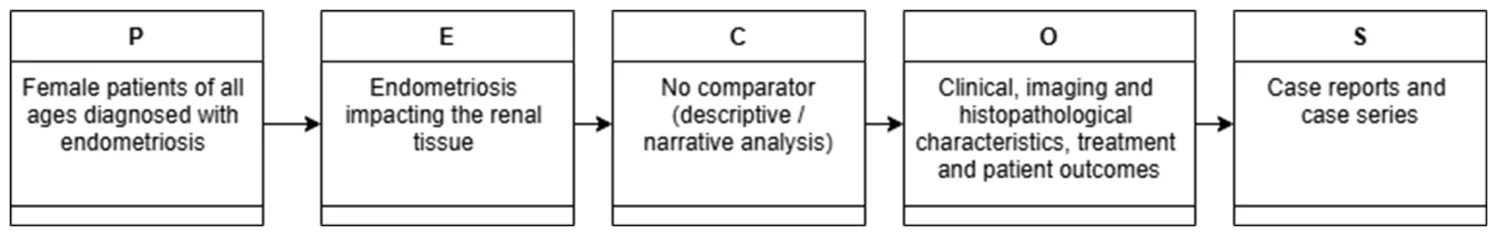
PECOS review framework.

Studies that satisfied the established inclusion and exclusion criteria outlined by the modified PECOS framework (“Population,” “Exposure,” “Comparison,” “Outcomes,” and “Study design”) were considered suitable for data extraction and subsequent analysis, as presented in Figure 1.

### 2.3. Data Extraction and Synthesis

We examined the literature with defined constructs, evaluating the studies to ascertain their alignment with these specified categories. This study, performed as a narrative review, employed an iterative and interpretative method for detecting new themes. The pertinent literature was initially examined for its conceptual compatibility with the review’s aims. The literature selection was determined by its pertinence to the clinical comprehension of the pathogenesis, diagnostic imaging, and therapy of documented cases of renal endometriosis. Throughout the review process, salient themes emerged through iterative readings and comparative analysis of findings across many sources. All iterative modifications during the review process were influenced by emergent themes and structures. The instances documented in the literature underscored particular diagnostic issues and management considerations that shaped the review’s focal points.

Due to the rarity of renal endometriosis, the available evidence is limited to case reports and small case series (Table 1), resulting in clinical and methodological heterogeneity and insufficient data for a systematic review. Consequently, formal systematic review frameworks, including PRISMA (Preferred Reporting Items for Systematic Reviews and Meta-Analyses) guidelines, were not incorporated.

As the aim was to provide a structured overview of the available case-based literature, a narrative review approach was applied. A structured literature search strategy and the PECOS framework were applied to enhance methodological transparency, reproducibility, and consistency.

## 3. Results

### 3.1. Study Characteristics

A total of 19 full-text available English-language publications of case reports were included in the in-depth analysis, and they are presented in Table 1 in chronological order. With the exception of one study that reported two cases, all other articles reported singleton case reports. In-depth analysis was performed on a total of 20 literature cases.

### 3.2. Overview of Clinical Findings

Noted condition predominantly impacted women of reproductive age, with an average age of 38.2, the youngest being 19 and the oldest, a solitary postmenopausal woman, aged 61.

**Figure 2:**
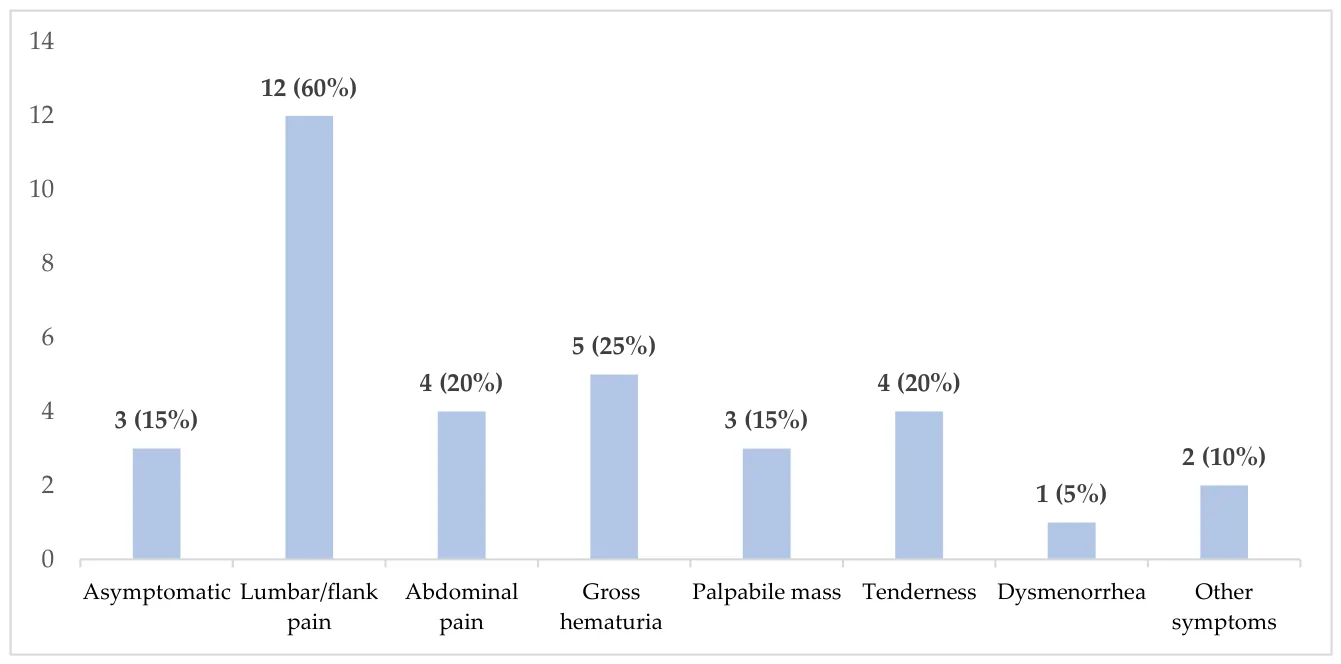
Summary of reported clinical features.

Among the evaluated patient symptoms and clinical characteristics (Figure 2), the most common presenting symptom was lumbar or flank pain, reported in 12 cases (60%) [17,18,19,23,24,25,26,28,29,30,32,34]. Gross hematuria was observed in five patients (25%) [19,24,28,29,31], while abdominal pain [20,21,22,33] and tenderness [23,24,26,34] were each noted in four cases (20%). A total of three patients (15%) [27,35] were asymptomatic at the time of diagnosis. Palpable mass was reported in three cases (15%) [18,23,24]. Dysmenorrhea was the least frequent symptom, present in one patient [24]. A total of three patients (15%) reported the exacerbation of symptoms during the menstrual cycle [24,26,31].

The occurrence of ovarian endometriosis lesions was documented in five (25%) instances [19,20,27,34]. Other reported comorbidities were limited to isolated cases of immune dysregulation disorders including diabetes mellitus [26,34], autoimmune thyroid disease [21], and antiphospholipid disorder [28]. Moreover, cases of malignant comorbidities were noted, including thyroid carcinoma [35], gastric carcinoma and melanoma [27]. Upon reporting, laboratory analyses were primarily marginal or within normal reference ranges. Renal function assessments (serum creatinine, blood urea nitrogen, and estimated glomerular filtration rate) and hemoglobin concentrations were predominantly within normal parameters [23,24,26,28,30,32,35].

### 3.3. Diagnosis—Computed Tomography as an Imaging Modality of Choice

The majority (16/20, 80%) of presented cases had CT in their diagnostic algorithm [20,21,23,24,25,26,27,28,29,31,32,33,34,35]. A total of 11 cases (55%) were reported on the right kidney, and 9 (45%) on the left. The majority of cases presented as one mass lesion (75%), while a small portion presented with multiple lesions (25% of cases). The mean largest diameter of all noted endometriosis lesions was 6.7 cm. Furthermore, it was significant that solitary lesions exhibited a greater size (with the mean diameter recorded at 6.2 cm, ranging from 1.2 to 13.5 cm), whereas multiple lesions were comparatively smaller (with a mean largest diameter of 1.2 cm, including reported millimetric and subcentimetric lesions, with a diameter range of 0.1 to 2 cm). One case presented itself as a subcapsular kidney hematoma, without definable lesion [23].

**Figure 3:**
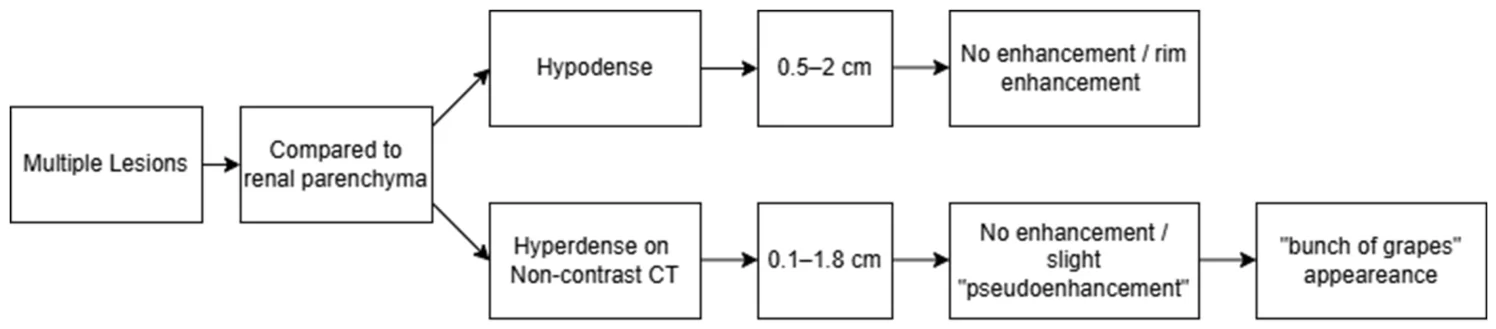
CT imaging flow diagram of multiple endometriosis lesions; “+C”—with contrast.

Multiple lesions were reported as hypo- [20,21,26] or hyperdense [27], exhibiting comparable maximum diameters (around 2 cm) and no notable post-contrast enhancement observed in the presented instances (Figure 3).

**Figure 4:**
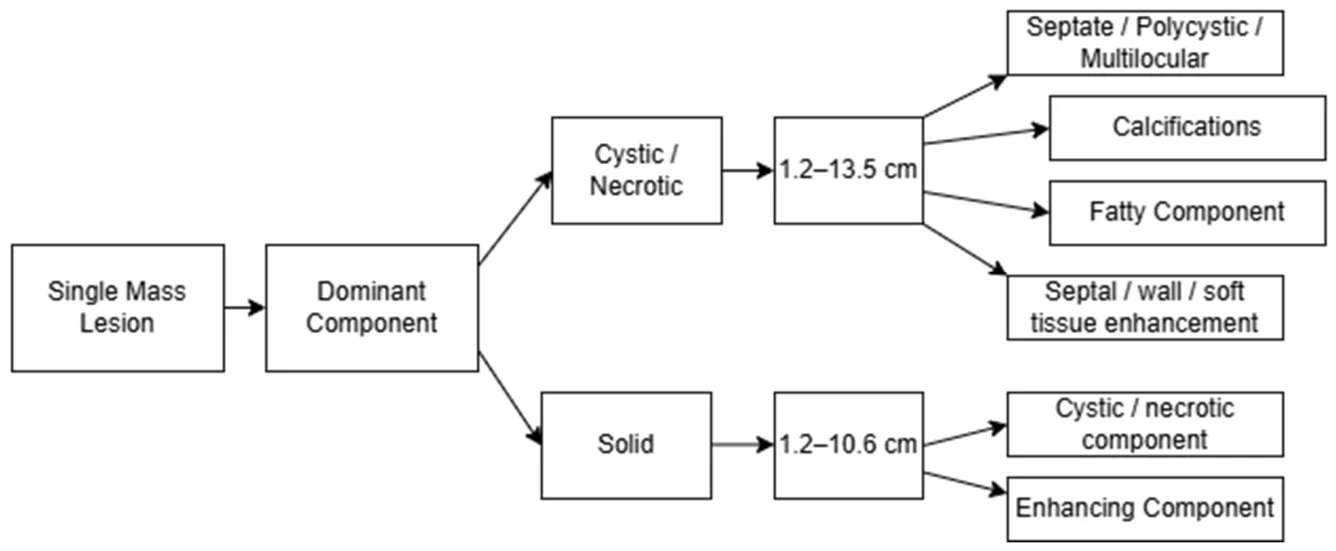
CT imaging flow diagram of one-mass endometriosis lesions; “+C”—with contrast.

Solitary mass lesions may be categorized via CT into two classifications according to the dominant component: cystic [22,24,25,31,32,34,35] and solid [28,29,33] (Figure 4).

In the context of cystic masses [24,25,31,32,34], where presented on unenhanced CT scans, some authors have noted hypo- to mildly hyperattenuating lesions on non-contrast CTs, exhibiting densities between 10 and 39 HU [25,34,35], mostly characterized as septated, multilocular formations [24,31]. On post-contrast CT scans, minor to moderate or partial enhancement was observed [25,31,32,34], along with foci of calcifications [32,34] and/or minimal intratumoral fat [24]. Moreover, extension of the mass into the renal pelvis has also been documented [32].

When it comes to solid lesion, highly attenuated, soft-tissue components were detected [28,29,33]. Intralesional alterations have also been documented, characterized by low-density core regions of necrosis or cystic transformation [33].

With noted features, in documented instances, the differential diagnosis could not rule out renal tumors, whether benign like AML [24] or malignant, and whether solid as RCC (renal cell carcinoma) [28,29] or cystic renal tumors (Bosniak III/IV) [25,31,32,34,35].

Surprisingly, MRI was utilized in only three reported cases [27,28,30], one of which involved a pregnant patient [30], thereby providing a specific indication for the use of this imaging modality. In the remaining cases [27,28], MRI was subsequently performed for additional lesion characterization, with no definitive diagnosis presented. An isolated case was characterized on both T1-weighted images (T1WI) and T2-weighted images (T2WI), presenting as numerous hyperintense lesions on fat-suppressed T1-WI, while presenting as hypointense on T2-WI, with a tendency to confluence [27]. Two further documented cases employing MRI presented T2-WI with hyperintense/cystic components [28,30]. No enhancement or adipose elements were documented in these instances.

### 3.4. Reported Management Choices and Outcomes

Given that the CT exhibited characteristics indicative of cystic or solid lesions, it could not definitively exclude malignant renal tumors. Noted CT findings were deemed sufficiently alarming to dictate further therapeutic decisions. In most instances, this necessitated a surgical procedure, primarily total nephrectomy in both cystic (54.5%) and solid lesions (62.5%). A small number of cases were eligible for partial nephrectomy (28.2% and 12.5%, respectively).

A renal mass biopsy was performed in 25% of cases as a diagnostic option for further clinical decision making, particularly useful as a diagnostic option in patients with significant comorbidities. One patient was put on hormonal manipulation with danazol and responding very well to this type of treatment with no surgery [21], while another received postoperative hormonal therapy [19,23]. Patients reported by Giambelluca et al. [27] were asymptomatic at the time of diagnosis and did not need any hormonal or surgical therapy for renal lesions, and were further followed-up. One work did not disclose the treatment of choice [20].

All presented cases made an uneventful, symptom-free recovery on reported controls, with no death cases (Table 1).

### 3.5. Histopathology Features

After FNA/biopsy or resection of the renal lesion, histopathology analysis was utilized in order to determine the diagnosis.

In majority of noted cases, the diagnosis was made based on detection endometrial epithelial cells [18,19,20,22,24,25,26,27,29,30,31,32,33,35], as well as endometrial stroma [18,22,24,26,27,29,30,31,32,35] (Table 2). In newer works (post 2013, a total of nine cases [24,26,27,28,30,31,32,35]), immunohistochemistry was implemented, with endometrial glands/epithelial cells and stromal cells positive for the estrogen receptor (ER) [26,27,30,31,35] and progesterone receptor (PR) [24,26,27,31,35]. One instance documented positive vimentin [31] or CK7 [30] in endometrial glands and stromal cells, alongside positive desmin, Melan-A and HMB-45 for smooth muscle metaplasia [28]. Stromal cells were mostly positive for CD10 when implemented [24,28,30,32,35].

## 4. Discussion

### 4.1. Patterns of Presentation in Reported Cases

As presented, renal endometriosis is a rather rare condition, making the identification of consistent clinical, imaging, and pathological patterns particularly challenging when exploring its presentation and underlying characteristics. The cyclic recurrence of symptoms associated with menstruation and the presence of gross hematuria, deemed pathognomonic for urinary tract endometriosis [36], was observed in merely a quarter of documented instances [19,24,28,29,31]. The remaining reported symptoms were largely nonspecific and generally suggestive of an underlying renal process and/or renal mass.

#### 4.1.1. Chronic Inflammation and Immune Dysregulation: Putative Drivers

Catamenial characteristics appear to be uncommon in these lesions [24,26,31], and only a small proportion of women reported dysmenorrhea [24] or concomitant ovarian endometriosis [19,20,27,34]. Because ectopic lesions may exhibit fluctuating hormonal responsiveness and activity, the lack of a distinct catamenial pattern does not rule out endometriosis. What can be deducted is that the reported symptomatology overall may suggest the presence of a renal mass associated with recurrent inflammation and progressive renal parenchyma destruction. Pro-inflammatory cytokines are key mediators of the chronic inflammatory environment that characterizes endometriosis and contributes to lesion progression. By impairing apoptotic mechanisms and promoting the survival and proliferation of ectopic endometrial tissue [37], they promote further invasion, fibrosis and adhesions in the endometriotic lesion, worsening the condition and prolonging the illness [38,39,40]. In compliance with the described pathogenesis, inflammatory alterations in endometriotic tissue may infiltrate the adjacent renal parenchyma, elucidating symptoms that manifest independently of hormonal variations. Chronic inflammation may eventually result in fibrosis, aligning with findings of chronic pyelonephritis in lesion-affected kidneys [30], local thinning of the renal cortex [31], as well as irregular, contracted [31] and scarred renal parenchyma [26], all potentially attributing to noted clinical findings. As the aforementioned processes are associated with immunological dysregulation, studies have indicated an association between endometriosis and several autoimmune disorders [41], along with other inflammatory conditions such as type 2 diabetes in specific subgroups [42] and atherosclerosis [43]. Similarly, numerous cases reported simultaneous autoimmune and inflammatory conditions [21,26,34,35]. Nonetheless, due to the limited number of instances, these data are merely descriptive and do not substantiate inferences about a correlation with renal endometriosis.

#### 4.1.2. Secondary Bacterial Infection and Dysbiosis: Emerging Hypotheses

Moreover, aforementioned mechanisms likely create a favorable environment for secondary bacterial infection, as different bacteria have been isolated from reported lesions such as *Citrobacter koseri* [26] and *Escherichia coli* [17]. Documented instances exhibited recurrent pyelonephritis [17], characterized as a catalyst for exacerbation of previously overlooked symptoms, ultimately presenting as acute-on-chronic pyelonephritis with abscess formation and areas of xanthogranulomatous pyelonephritis [26]. Typically, subsequent infections resulting from endometriomas are exceedingly rare, and their underlying mechanisms remain poorly understood. An alteration in the immunological milieu of the endometrial glands and stroma could potentially lead to endometrioma infection, as bacterial proliferation is facilitated by the accumulation of blood in a cystic region and the cyst’s delicate wall [44]. Certain studies also suggest that endometriosis may be partially caused by bacterial infections, particularly those of the genus *Fusobacterium* [45], as dysbiosis of the female reproductive tract, creating dysregulated immune homeostasis supporting onset and progression of endometriosis [46].

### 4.2. Imaging—Definitive Patterns or a Warning Sign for Surgery?

On imaging, kidney endometriosis was slightly more commonly reported on the right side. This deviates from the more common occurrence of left-sided ureteric endometriosis, attributed to the notion that the sigmoid colon’s left position creates a microenvironment surrounding the left adnexa, promoting interaction between endometrial cells and the regional peritoneum [47,48]. These results may indicate that remote endometriosis is considerably more intricate than the concept of “regurgitation” and arises thru several pathways [49]. However, this conclusion cannot be definitively established based on the current evidence. The restricted sample size and the marginally higher prevalence of lesions on the right side render the observed distribution inadequate for drawing definitive conclusions about the underlying etiology. Additional research involving bigger cohorts is required to elucidate the causes of remote endometriosis.

In presented cases, a variety of imaging modalities were used, with CT being the most crucial when making clinical decisions. Overviewing reported CT imaging features, but it did not reveal any particularly distinctive patterns, nor a specific CT characteristic that may indicate endometriosis. Both multiple and solitary masses were reported, with solid and cystic lesions described in almost equal proportions. Single mass-forming endometriosis lesions were indistinguishable from more complicated and malicious disorders such Bosniak III/IV cysts and various RCC forms [28,29,32,33,35]. Hemorrhagic cysts were the primary benign differential diagnosis, with non-contrast high attenuation and no substantial postcontrast enhancement, but it was not possible to rule out other more serious growths [27].

One case reported typical MRI features of endometriosis [27]. As a result of recurrent hemorrhaging, increased viscosity resulting from the iron overload and high protein concentrations displays the distinctive pattern on T2WI of the endometrial cysts, referred to as “T2 shading,” characterized by a hypointense signal on T2WI and a hyperintense signal on T1WI, with a diagnostic specificity exceeding 90% for endometriosis [50]. In rare cases, pelvic endometriomas may appear T2-hyperintense, depending on protein and hemosiderin concentrations [51]. However, even though limited in the literature, endometriotic lesions have previously been reported as cystic (T2WI hyperintense) in non-pelvic endometriosis, particularly in other parenchimatous organs such as pancreas [52] and liver [53]. Unlike deep infiltrating endometriosis, with signal intensity close to that of pelvic muscle on T1- and T2-WI due to extensive fibrosis [54], parenchymal extra-pelvic endometriomas are often T2 hyperintense, due to their primarily cystic and hemorrhagic nature, which could be attributed to repeated hemorrhage and inflammation in a localized, tissue-dense region.

As CT is nonspecific for diagnosing endometriosis due to low tissue characterization capabilities [55], with features overlapping with various renal diseases, MRI may assist in differential diagnosis in specific instances. Cystic RCCs and multilocular cystic renal neoplasms typically exhibit nodular and thick septal enhancement, which is the most sensitive indicator for distinguishing cystic RCC from complex benign lesions [56]. In the absence of contrast enhancement, the T1WI lesion-to-muscle signal-intensity ratio has been identified as an effective metric for differentiating slightly hyperintense RCCs from hemorrhagic cysts, with the ratio being much lower in RCCs compared to benign hemorrhagic renal cysts [57]. Furthermore, hemorrhagic renal cysts may resemble endometriosis due to T1 hyperintensity, but catamenial symptoms are absent. The presence of T2 shading sign in the context of severe, persistent bleeding would be very indicative of endometriosis, as RCC intratumor hemorrhage manifesting as patchy or punctate [58]. Although a minor focus of fat was identified in one instance of renal endometriosis, it represents a significant differential that cannot be accurately distinguished from hypovascular RCCs or other diseases, necessitating surgical intervention or biopsy for distinction [59].

Despite comprehensive research on morphological and quantitative parameters in traditional imaging, no CT or MR imaging modalities can consistently differentiate between solid benign and malignant kidney malignancies [60]. Despite the utilization of MRI, inconsistencies in renal mass characterization continue to be reported, indicating the need for better diagnostic methods [61]. CT scans with contrast facilitate the differentiation between solid and cystic renal lesions, highlighting concerning characteristics such as enhancing soft tissue or hemorrhagic elements that are readily identifiable [55,62]. Therefore, a larger mass detected on CT may not be followed by MRI when the CT findings are considered sufficiently concerning to directly guide further management, particularly if surgical intervention is already indicated based on clinical findings. Pretreatment percutaneous biopsy can possibly decrease the number of unnecessary surgeries for benign disease and assist the urologist in clinical decision-making [62].

The histopathological diagnosis of endometriosis is generally straightforward, involving the detection of endometrial epithelial and stromal cells. Nonetheless, other diagnostic difficulties may arise from changes in identified foci lacking their glandular or stromal components [63,64]. The stromal component may be concealed or diminished by the infiltration of other cells, such as pigmented histiocytes [20,22,27,30] or smooth muscle metaplasia [28]. Inflammatory alterations around endometriosis can obscure histological interpretations [63], as several studies have documented renal tubular epithelial cells and tissue [20,32] including infections within endometriotic cysts, as previously noted.

### 4.3. Treatment, Management, and Outcomes

With the extreme rarity of kidney involvement, we underscore the necessity for a multidisciplinary approach, as per European Society of Human Reproduction and Embryology (ESHRE) Endometriosis Guidelines [65]. In cases where extra-pelvic endometriosis is considered in the differential diagnosis, the involved team primarily consists of gynecologists, urologists, and radiologists. The principal specialists comprise gynecologists and urologists to diagnose and manage urinary tract involvement. Additional specialists may encompass radiologists who appropriately interpret imaging studies and facilitate intervention. The superior efficacy of MRI in detecting and mapping lesions has been emphasized in cases of endometriosis inside complicated anatomical locations, especially the urinary system, and therefore should be utilized in further cases [66].

As per their size (average of 6.7 cm) and RCC-mimicry (both solid and cystic), surgery is the general treatment approach. The limited use of biopsy in reported renal lesions likely reflects contemporary clinical practice, in which management decisions are frequently guided by imaging characteristics and surgical respectability, as well as patient factors and preferences [67], rather than preoperative tissue confirmation. Surgical resection appears to offer safe and durable symptom relief, as per other locations of the urinary tract endometriosis [36]. Renal biopsies have been recommended in circumstances when they could alter treatment (with hormone therapy or regular follow-ups). If surgery is not a therapeutic option, pharmacological treatment is believed to offer short-term symptom relief but is unable to adequately treat the underlying illness, with a high chance of recurrence after stopping treatment [68]. Post-surgical adjuvant therapy, namely a brief regimen of hormonal treatment, has been recommended to enhance surgical efficacy and mitigate recurrences of endometriosis, particularly deep peritoneal endometriosis [69].

Finally, despite the absence of reported recurrences in the analyzed cases, the restricted sample size and inconsistent follow-up periods hinder conclusive inferences when it comes to treatment outcomes. Although hormone therapy is the primary method for managing postoperative symptoms and mitigating immediate pain while decreasing recurrence risk [65], its efficacy after nephrectomy for isolated renal endometriosis remains ambiguous due to insufficient evidence. The sole legitimate inference from the provided data is that healthcare professionals ought to adopt a multidisciplinary approach to decision-making, taking into account individual preferences, side effects, efficacy, costs, and availability when choosing between hormone therapies and surgical interventions for endometriosis-related pain, as per ESHRE Endometriosis Guidelines [65].

## 5. Conclusions

The combination of nonspecific symptoms, the lack of a clearly reported association with the menstrual cycle, and the rare occurrence of dysmenorrhea and concomitant ovarian endometriosis make the diagnosis of these lesions exceptionally challenging, with no distinct endometriosis-specific clinical pattern emerging. Moreover, no distinctive CT imaging characteristics were reported, as both solid and cystic lesions were almost equally reported, with calcifications and different enhancing patterns. Therefore, renal endometriosis is difficult to present as a differential diagnosis and the final diagnosis ultimately relies on the pathohistologic findings and detection of endometrial glands and stromal cells. Consequently, in reproductive-age women presenting with such symptoms in the absence of specific clinical or imaging findings suggestive of another urogenital site pathology, endometriosis should be considered in the differential diagnosis, with multidisciplinary decision-making approach in disease management.

## Figures and Tables

**Table 1:** Narrative summary of compiled study characteristics.

First Author (Year, Country)	Age	Short Lesion Description	Significant Clinical and Laboratory Findings; Comorbidities	Differential/Initial Diagnosis	Management	Outcome (Months on Control *)
Gaupera et al. (1977, Norway) [17]	23	A spherical fluctuating, protruding mass	Lumbar pain, tenderness	Clear cell carcinoma	Nephrectomy	Not specified
Bazaz-Malik et al. (1980, India) [18]	40	Two cystic masses in the cortex	Lumbar pain, palpable mass	Hydatid cyst	Nephrectomy	Not specified
Hellber et al. (1991, Sweden) [19]	25	Cystic tumor at the upper pole of the left kidney	Severe back pain, gross hematuria	Renal tumor	Not specified (biopsy only)	Symptom-free (8m)
Gupta et al. (2004, India) [20]	40	Multiple small hypodense foci in the right kidney	Abdominal pain	Not specified	Not specified (biopsy only)	Not specified
Dutta et al. (2006, India) [21]	38	Enlarged right kidney with multiple focal hypodense lesions	Abdominal pain; autoimmune thyroid disease	Not specified	Not specified (biopsy only)	Responding well to treatment
Yaqub et al. (2008, Pakistan) [22]	25	Large cyst occupying the left paracolic gutter	Lower abdominal pain	Mesenteric cyst, RP mass	Cystectomy/Nephrectomy	Uneventful recovery after surgery
Dirim et al. (2009, Turkey) [23]	46	Renal subcapsular hematoma originating from the lower pole of the left kidney	Lumbar pain and mass, tenderness, normal laboratory findings	Subcapsular hematoma	Hematoma drainage	No pathology (1m)
Jiang et al. (2013, Taiwan) [24]	42	Septate, encapsulated right renal tumor	Lumbar pain, palpable mass, hematuria, dysmenorrhea, normal lab. findings	Angiomyolipoma	Nephrectomy	No lesion or hematuria (10m)
Yang et al. (2015, China) [25]	37	An inhomogeneous mass with calcifications, necrosis and cystic change	Right lower back pain aggravated by menstrual periods	Cystic RCC	Nephrectomy	Not specified
Cheng et al. (2015, Taiwan) [26]	53	Atrophic right kidney, perirenal abscess and severe adhesions	Intermittent flank pain, tenderness, normal BUN and creatinine; DM	Acute-on-chronic pyelonephritis	Nephrectomy	Symptom-free (1m)
Giambelluca et al. (2017, Italy) [27]	40, 39	Multiple hyperattenuating contiguous nodular lesions (“bunch of grapes”)	Incidental finding (evaluation for gastric carcinoma and melanoma)	Hemorrhagic cysts	Not specified (biopsy only)	No clinical or imaging changes (6m)
Badri et al. (2018, USA) [28]	45	Heterogeneous, partially enhancing, cystic loculated mass	Intermittent flank pain, gross hematuria, normal laboratory findings	RCC, Benign renal mass	Partial nephrectomy	Symptom-free on last visit
Tabakin et al. (2018, USA) [29]	41	Solid, enhancing left kidney mass	Lumbar pain, gross hematuria	RCC	Nephrectomy	No recurrence (12m)
Umair et al. (2020, Pakistan) [30]	30	Solid right renal mass	Lumbar pain, normal laboratory findings	Not specified	Nephrectomy	Not specified
Yang et al. (2021, China) [31]	48	A well-demarcated septated, polycystic tumor	Intermittent gross hematuria	Cystic renal tumor	Partial nephrectomy	No pathology (3m)
Nawaz et al. (2022, Pakistan) [32]	30	Multilocular cystic mass with enhancing component	Flank pain, normal laboratory findings	Cystic renal tumor/Bosniak IV	Nephrectomy	Uneventful recovery
Chen et al. (2023, China) [33]	19	Hypodense mass in the left ectopic kidney	Abdominal pain	Not specified	Partial nephrectomy	Not specified
Healey et al. (2023, USA) [34]	61	Complex cystic mass with calcifications	Flank pain, dysuria (hematuria and leukocyturia), nausea, fever; DM	Bosniak III	Nephrectomy	Uneventful recovery
Katsikatsos et al. (2024, Greece) [35]	42	Slightly enhanced mass on the left hypoplastic kidney	Asymptomatic, normal GFR; thyroid cancer	Bosniak IV	Nephrectomy	Symptom-free on last visit

* Months of the last noted posttreatment/postoperative controls were noted where specified. USA—United States of America.; RCC—Renal cell carcinoma; RP—retroperitoneal; BUN—blood urea nitrogen; DM—diabetes mellitus; GFR—glomerular filtration rate.

**Table 2:** Overview of histopathology and immunohistochemistry features.

Tissue Type	Cell Type	Immunology	Reference
Endometrial tissue	Endometrial epithelial cells and stroma	ER+ PR+ Vimentin+	[26,27,30,31,35]
			[24,26,27,31,35]
			[31]
	Endometrial epithelial cells/glands	CK7	[30]
	Endometrial stromal cells	CD10+	[24,28,30,32,35]
Other	Smooth muscle metaplasia	desmin, Melan-A, HMB-45+	[28]
	Hemorrhage, hemosiderin Macrophages	Not Applicable	[20,22,27,30]

ER, estrogen receptor; PR, progesterone receptor; CK7, cytokeratin 7; CD10, cluster of differentiation 10; Melan-A, melanocytic differentiation antigen; HMB-45, human melanoma black 45. “+” indicates positive immunohistochemical staining.

## Data Availability

No new data were created or analyzed in this study. Data sharing is not applicable to this article.

## Abbreviations

The following abbreviations are used in this manuscript:

CT	Computed Tomography
MRI	Magnetic Resonance Imaging
USA	United States of America
AML	Angiomyolipoma
RCC	Renal cell carcinoma
ER	Estrogen receptor
PR	Progesterone receptor
CK7	cytokeratin 7
CD10	Cluster of differentiation 10
Melan-A	Melanocytic differentiation antigen
HMB-45	Human melanoma black 45

## References

[1] Parasar P., Ozcan P., Terry K.L. (2017). Endometriosis: Epidemiology, Diagnosis and Clinical Management. Curr. Obstet. Gynecol. Rep..

[2] Smolarz B., Szyłło K., Romanowicz H. (2021). Endometriosis: Epidemiology, Classification, Pathogenesis, Treatment and Genetics (Review of Literature). Int. J. Mol. Sci..

[3] Saunders P.T.K., Horne A.W. (2025). Endometriosis: New Insights and Opportunities for Relief of Symptoms. Biol. Reprod..

[4] Zhang J., Pang M., Li L., Guo C. (2025). Global, Regional, and National Burden of Endometriosis among Women of Reproductive Age, 1990–2021: Insights from the Global Burden of Disease Study 2021. PLoS ONE.

[5] Lee H.J., Park Y.M., Jee B.C., Kim Y.B., Suh C.S. (2015). Various Anatomic Locations of Surgically Proven Endometriosis: A Single-Center Experience. Obstet. Gynecol. Sci..

[6] Andres M.P., Arcoverde F.V.L., Souza C.C.C., Fernandes L.F.C., Abrão M.S., Kho R.M. (2020). Extrapelvic Endometriosis: A Systematic Review. J. Minim. Invasive Gynecol..

[7] Piriyev E., Schiermeier S., Römer T. (2025). Bladder Endometriosis: Diagnostic, Therapy, and Outcome of a Single-Center Experience. Diagnostics.

[8] Leonardi M., Espada M., Kho R.M., Magrina J.F., Millischer A.-E., Savelli L., Condous G. (2020). Endometriosis and the Urinary Tract: From Diagnosis to Surgical Treatment. Diagnostics.

[9] Fleischer K., Bachi A., Kam J., Narayanan P., Nair R., Khazali S. (2024). Bladder Endometriosis: What Do We Know and What Is Left to Find out? A Narrative Review. Best Pract. Res. Clin. Obstet. Gynaecol..

[10] Al Ayoubi O., Aldakak M.A., Tannous G., Mhimid A., Awad M.A., Alia L. (2025). Ureteral Endometriosis Presenting as Recurrent Hydronephrosis: A Case Report. Res. Rep. Urol..

[11] Van Ligten M.J., Sobel T., Shihab S., Urumov A., Adler C.R., Martini W.A. (2024). Obstruction from Endometriosis Causing Hydronephrosis and Complex Renal Pelvis Rupture: A Case Report. Case Rep. Women’s Health.

[12] Li J., Bai J., Wang H., Chen B. (2024). A Case Report of Ureteral Endometriosis with Severe Hydronephrosis. Urol. Case Rep..

[13] Kolodziej A., Krajewski W., Dolowy L., Hirnle L. (2015). Urinary Tract Endometriosis. Urol. J..

[14] Charatsi D., Koukoura O., Ntavela I.G., Chintziou F., Gkorila G., Tsagkoulis M., Mikos T., Pistofidis G., Hajiioannou J., Daponte A. (2018). Gastrointestinal and Urinary Tract Endometriosis: A Review on the Commonest Locations of Extrapelvic Endometriosis. Adv. Med..

[15] Marshall V.F. (1943). The Occurrence of Endometrial Tissue in the Kidney. Case Report and Discussion. J. Urol..

[16] Hajdu S.I., Koss L.G. (1970). Endometriosis of the Kidney. Am. J. Obstet. Gynecol..

[17] Gauperaa T., Stalsberg H. (1977). Renal Endometriosis: A Case Report. Scand. J. Urol. Nephrol..

[18] Bazaz-Malik G., Saraf V., Rana B.S. (1980). Endometrioma of the Kidney: Case Report. J. Urol..

[19] Hellberg D., Fors B., Bergqvist C. (1991). Renal Endometriosis Treated with a Gonadotrophin Releasing Hormone Agonist. Case Report. BJOG.

[20] Gupta K., Rajwanshi A., Srinivasan R. (2005). Endometriosis of the Kidney: Diagnosis by Fine-Needle Aspiration Cytology. Diagn. Cytopathol..

[21] Dutta P., Bhat M.H., Bhansali A., Kumar V. (2006). A Young Woman with Endometriosis of Kidney. Saudi Med. J..

[22] Yaqub U., Hassan S., Yusaf Z., Yusuf A.W. (2008). Endometriosis in the Renal Area. J. Coll. Physicians Surg. Pak..

[23] Dirim A., Celikkaya S., Aygun C., Caylak B. (2009). Renal Endometriosis Presenting with a Giant Subcapsular Hematoma: Case Report. Fertil. Steril..

[24] Jiang Y.-H., Kuo H.-C., Hsu Y.-H. (2013). Renal Endometriosis Mimicking an Angiomyolipoma. Urol. Sci..

[25] Yang J., Song R., Xu C., Zhang S., Zhang W. (2015). Renal Endometriosis Tends to Be Misdiagnosed as Renal Tumor: A Rare Case Report. Int. Surg..

[26] Cheng C.-H., Kuo H.-C., Su B. (2015). Endometriosis in a Kidney with Focal Xanthogranulomatous Pyelonephritis and a Perinephric Abscess. BMC Res. Notes.

[27] Giambelluca D. (2017). Renal Endometriosis Mimicking Complicated Cysts of Kidney: Report of Two Cases. GHIR.

[28] Badri A.V., Jennings R., Patel P., Eun D.D. (2018). Renal Endometriosis: The Case of an Endometrial Implant Mimicking a Renal Mass. J. Endourol. Case Rep..

[29] Tabakin A.L., Dutton J.L., Fatima A., Sadimin E., Jang T.L. (2018). Polypoid Endometriosis Presenting as a Renal Cortical Tumor. Urology.

[30] Umair M., Nawaz M., Murtaza B., Ali A., Khan F.B., Wahab A.U. (2020). Renal Endometriosis Mimicking a Renal Tumor in a Pregnant Patient. Urol. Case Rep..

[31] Yang Y., Zhao X., Huang Y. (2021). Renal Endometriosis Mimicking Cystic Renal Tumor: Case Report and Literature Review. Front. Med..

[32] Nawaz M.M., Masood Y., Usmani A.S., Basheer M.I., Sheikh U.N., Mir K. (2022). Renal Endometriosis: A Benign Disease with Malignant Presentation. Urol. Case Rep..

[33] Chen M., Yu Y., Zhao X. (2023). Endometriosis in an Ectopic Kidney: A Rare Case Report and Literature Review. BMC Women’s Health.

[34] Healey K.D., Herson A.B., Phrathep D.D., Schwarz C., Ramos C.E., Rifai A.O. (2023). Renal Endometriosis in a Postmenopausal Female Mimics Renal Cell Carcinoma. Cureus.

[35] Katsikatsos P., Douroumis K., Goutas D., Gakiopoulou H., Anastasiou P., Anastasiou I. (2024). Renal Endometriosis Mimics Renal Cell Carcinoma in a Hypoplastic Kidney: A Case Report. Cureus.

[36] Sherman A.K., MacLachlan L.S. (2022). A Review of Urinary Tract Endometriosis. Curr. Urol. Rep..

[37] Lamceva J., Uljanovs R., Strumfa I. (2023). The Main Theories on the Pathogenesis of Endometriosis. Int. J. Mol. Sci..

[38] Machairiotis N., Vasilakaki S., Thomakos N. (2021). Inflammatory Mediators and Pain in Endometriosis: A Systematic Review. Biomedicines.

[39] Rathod S., Shanoo A., Acharya N. (2024). Endometriosis: A Comprehensive Exploration of Inflammatory Mechanisms and Fertility Implications. Cureus.

[40] Oală I.E., Mitranovici M.-I., Chiorean D.M., Irimia T., Crișan A.I., Melinte I.M., Cotruș T., Tudorache V., Moraru L., Moraru R. (2024). Endometriosis and the Role of Pro-Inflammatory and Anti-Inflammatory Cytokines in Pathophysiology: A Narrative Review of the Literature. Diagnostics.

[41] Shigesi N., Kvaskoff M., Kirtley S., Feng Q., Fang H., Knight J.C., Missmer S.A., Rahmioglu N., Zondervan K.T., Becker C.M. (2019). The Association between Endometriosis and Autoimmune Diseases: A Systematic Review and Meta-Analysis. Hum. Reprod. Update.

[42] Farland L.V., Degnan W.J., Harris H.R., Tobias D.K., Missmer S.A. (2021). A Prospective Study of Endometriosis and Risk of Type 2 Diabetes. Diabetologia.

[43] Poznyak A.V., Sukhorukov V.N., Sobenin I.A., Ofitserov V.P., Golovyuk A.L., Serdyukov D.Y., Postnov A.Y., Orekhov A.N. (2024). Exploring the Link between Chronic Inflammation and Atherosclerosis-Endometriosis Association. J. Angiother..

[44] Siati A., El Jawhari M., Dehayni M. (2024). An Uncommon Case of an Infected Ovarian Endometrioma. Int. J. Surg. Case Rep..

[45] Muraoka A., Suzuki M., Hamaguchi T., Watanabe S., Iijima K., Murofushi Y., Shinjo K., Osuka S., Hariyama Y., Ito M. (2023). *Fusobacterium* Infection Facilitates the Development of Endometriosis through the Phenotypic Transition of Endometrial Fibroblasts. Sci. Transl. Med..

[46] Yuanyue L., Qian H., Ling L., Liufeng Y., Jing G., Xiaomei W. (2025). Impact of Gut Microbiota on Endometriosis: Linking Physical Injury to Mental Health. Front. Cell. Infect. Microbiol..

[47] Alaoui Mhammedi W., Ouraghi A., Irzi M., El Moudane A., Mokhtari M., Barki A. (2022). Ureteral Endometriosis Presenting as Left Ureteral Obstruction: A Case Report. Cureus.

[48] Bahall V., Hosein Y., Konduru S., Barrow M. (2021). Isolated Intrinsic Ureteral Endometriosis: A Rare Presentation of Ureteral Obstruction. Cureus.

[49] Jiang L., Yan Y., Liu Z., Wang Y. (2016). Inflammation and Endometriosis. Front. Biosci. (Landmark Ed.).

[50] Tran-Harding K., Nair R.T., Dawkins A., Ayoob A., Owen J., Deraney S., Lee J.T., Stevens S., Ganesh H. (2018). Endometriosis Revisited: An Imaging Review of the Usual and Unusual Manifestations with Pathological Correlation. Clin. Imaging.

[51] Ruaux E., Nougaret S., Gavrel M., Charlot M., Devouassoux-Shisheboran M., Golfier F., Thomassin-Naggara I., Rousset P. (2024). Endometriosis MR Mimickers: T1-Hyperintense Lesions. Insights Imaging.

[52] Paramythiotis D., Syrnioti A., Tsavdaris D., Smprini A., Mekras A., Apostolidis A., Cheva A. (2025). Pancreatic Endometriosis Coexisting with a Splenic Mesothelial Cyst: A Rare Case Report and Review of the Literature. Diseases.

[53] McCall J., Busca A., Gilbert S., Williams E., Horwood G., Singh S.S. (2024). Liver Endometrioma: A Rare Extrapelvic Site of Endometriosis Causing Catamenial Right Shoulder Pain. Am. J. Obstet. Gynecol..

[54] Bazot M., Darai E., Hourani R., Thomassin I., Cortez A., Uzan S., Buy J.-N. (2004). Deep Pelvic Endometriosis: MR Imaging for Diagnosis and Prediction of Extension of Disease. Radiology.

[55] Ghosh S., Alhamshari A., Prajapati P., Nakrour N., Carnelli C., Kilcoyne A., Harisinghani M.G., Tsai L.L., Catalano O.A., Kambadakone A. (2025). Role of Computed Tomography in Imaging of Endometriosis. Abdom. Radiol..

[56] Freire M., Remer E.M. (2009). Clinical and Radiologic Features of Cystic Renal Masses. Am. J. Roentgenol..

[57] McKee T.C., Dave J., Kania L., Karajgikar J., Masarapu V., Deshmukh S., Roth C. (2019). Are Hemorrhagic Cysts Hyperintense Enough on T1-Weighted MRI to Be Distinguished from Renal Cell Carcinomas? A Retrospective Analysis of 204 Patients. Am. J. Roentgenol..

[58] Xing W., He X., Kassir M.A., Chen J., Ding J., Sun J., Hu J., Zhang Z., Haacke E.M., Dai Y. (2013). Evaluating Hemorrhage in Renal Cell Carcinoma Using Susceptibility Weighted Imaging. PLoS ONE.

[59] Labra A., Schiappacasse G., Constenla D., Cristi J. (2025). Renal Angiomyolipomas: Typical and Atypical Features on Computed Tomography and Magnetic Resonance Imaging. World J. Radiol..

[60] Kang S.K., Huang W.C., Pandharipande P.V., Chandarana H. (2014). Solid Renal Masses: What the Numbers Tell Us. AJR Am. J. Roentgenol..

[61] Lee C.U., Kim J.H., Kim G., Jeon J., Kim J.W., Lee Y.S., Min K., Tae J.H., Choi S.Y., Chang I.H. (2025). Impact of Tumor Size and Sex on Diagnostic Discrepancies in Renal Mass Imaging. Sci. Rep..

[62] Pallwein-Prettner L., Flöry D., Rotter C.R., Pogner K., Syré G., Fellner C., Frauscher F., Aigner F., Krause F.S., Fellner F. (2011). Assessment and Characterisation of Common Renal Masses with CT and MRI. Insights Imaging.

[63] Clement P.B. (2007). The Pathology of Endometriosis: A Survey of the Many Faces of a Common Disease Emphasizing Diagnostic Pitfalls and Unusual and Newly Appreciated Aspects. Adv. Anat. Pathol..

[64] McCluggage W.G. (2020). Endometriosis-related Pathology: A Discussion of Selected Uncommon Benign, Premalignant and Malignant Lesions. Histopathology.

[65] Becker C.M., Bokor A., Heikinheimo O., Horne A., Jansen F., Kiesel L., King K., Kvaskoff M., Nap A., Petersen K. (2022). ESHRE Guideline: Endometriosis. Hum. Reprod. Open.

[66] Fatehchehr S., Jiang M., Nasab S. (2026). Urinary Tract Endometriosis: Systematic Review. J. Endometr. Pelvic Pain Disord..

[67] McAlpine K., Breau R.H., Stacey D., Knee C., Jewett M.A.S., Violette P.D., Richard P.O., Cagiannos I., Morash C., Lavallée L.T. (2020). Shared Decision-Making for the Management of Small Renal Masses: Development and Acceptability Testing of a Novel Patient Decision Aid. Can. Urol. Assoc. J..

[68] Thanasa E., Thanasa A., Kamaretsos E., Kontogeorgis G., Paraoulakis I., Thanasas I. (2024). Surgical Treatment of a Rare Case of Extrapelvic Endometriosis in the Rectus Abdominis Muscles with Negative Imaging Findings: A Case Report and Mini Literature Review. Cureus.

[69] Somigliana E., Busnelli A., Benaglia L., Viganò P., Leonardi M., Paffoni A., Vercellini P. (2017). Postoperative Hormonal Therapy after Surgical Excision of Deep Endometriosis. Eur. J. Obstet. Gynecol. Reprod. Biol..

